# Author Correction: Elevated complement mediator levels in endothelial-derived plasma exosomes implicate endothelial innate inflammation in diminished brain function of aging humans

**DOI:** 10.1038/s41598-022-05977-3

**Published:** 2022-02-01

**Authors:** Fanny M. Elahi, Danielle Harvey, Marie Altendahl, Nivetha Brathaban, Nicole Fernandes, Kaitlin B. Casaletto, Adam M. Staffaroni, Pauline Maillard, Jason D. Hinman, Bruce L. Miller, Charles DeCarli, Joel H. Kramer, Edward J. Goetzl

**Affiliations:** 1 Memory and Aging Center, Department of Neurology, University of California, San Francisco, San Francisco, CA USA; 2 Department of Public Health Sciences, University of California, Davis, Davis, CA USA; 3 Department of Neurology and Center for Neuroscience, University of California, Davis, Davis, CA USA; 4 Department of Neurology, University of California, Los Angeles, Los Angeles, CA USA; 5 Department of Medicine, University of California, San Francisco, San Francisco, CA USA; 6 Jewish Home of San Francisco, San Francisco, CA USA; 7 Geriatric Research Center, 1719 Broderick St., San Francisco, CA USA

Correction to: *Scientific Reports* https://doi.org/10.1038/s41598-021-91759-2, published online 10 August 2021

The original version of this Article contained errors in the presentation of the author names Fanny M. Elahi, Danielle Harvey, Marie Altendahl, Nivetha Brathaban, Nicole Fernandes, Kaitlin B. Casaletto, Adam M. Staffaroni, Pauline Maillard, Jason D. Hinman, Bruce L. Miller, Charles DeCarli, Joel H. Kramer and Edward J. Goetzl, which were incorrectly given as F. M. Elahi, D. Harvey, M. Altendahl, N. Brathaban, N. Fernandes, K. B. Casaletto, A. M. Stafaroni, P. Maillard, J. D. Hinman, B. L. Miller, C. DeCarli, J. H. Kramer and E. J. Goetzl.

Furthermore, Affiliation 7 ‘Department of Neurology, Memory and Aging Center, University of California, San Francisco, 675 Nelson Rising Lane, Suite 190, San Francisco, CA 94158, USA’ was a duplicate of Affiliation 1 ‘Memory and Aging Center, Department of Neurology, University of California, San Francisco, San Francisco, CA, USA’. The duplicate has been removed.

The original Article and its accompanying Supplementary Information files have been corrected.

