# Supplementary material for: Elevated complement mediator levels in endothelial-derived plasma exosomes implicate endothelial innate inflammation in diminished brain function of aging humans
Source: Sci Rep. 2021 Aug 10;11:16198. doi: 10.1038/s41598-021-91759-2 (PMC8355229; doi:10.1038/s41598-021-91759-2)
Supplement: Supplementary file 1 — Supplementary Information 1. [file 41598_2021_91759_MOESM1_ESM.pdf]

Title: Elevated complement mediator levels in endothelial-derived plasma exosomes implicate endothelial innate inflammation in diminished brain function of aging humans

Authors: Fanny M. Elahi[1*], Danielle Harvey[2], Marie Altendahl[1], Nivetha Brathaban[1], Nicole Fernandes[1], Kaitlin B. Casaletto[1], Adam M. Staffaroni[1], Pauline Maillard[3], Jason D. Hinman[4], Bruce L. Miller[1], Charles DeCarli[3], Joel H. Kramer[1], Edward J. Goetzl[5,6,7*]

[1]Memory and Aging Center, Department of Neurology, University of California, San Francisco. San Francisco, CA, USA

[2]Department of Public Health Sciences, University of California, Davis. Davis, CA, USA

[3]Department of Neurology and Center for Neuroscience, University of California, Davis. Davis, CA, USA

[4]Department of Neurology, University of California, Los Angeles. Los Angeles, CA, USA

[5]Department of Medicine, University of California, San Francisco. San Francisco, CA, USA

[6]Jewish Home of San Francisco. San Francisco, CA, USA

[7]Geriatric Research Center, 1719 Broderick St., San Francisco, CA, USA

Keywords: cerebral small vessel disease; exosomes; innate immunity; inflammation; complement factors; biomarkers; white matter; neurodegeneration.

[*]Corresponding Authors:
Fanny M. Elahi, MD PhD
675 Nelson Rising Lane, Suite 190, San Francisco, CA, 94158
Emails: fanny.elahi@ucsf.edu and elahilab15@gmail.com

Edward J. Goetzl, MD
Geriatric Research Center, 1719 Broderick St., San Francisco, CA 94115
E-mail: edward.goetzl@ucsf.edu

Supplemental Figure 1 | Complement Pathways

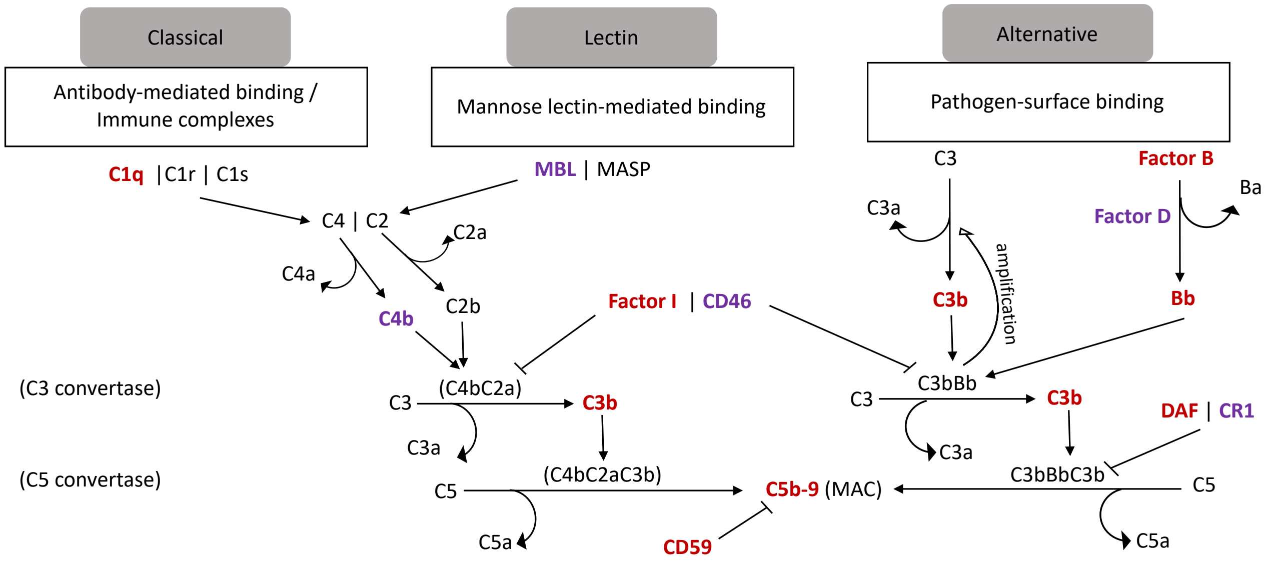

In red bold complement factors with significantly different levels between groups, and in purple bold complement factors not found to have significantly different levels between groups. The extreme inflammatory consequences of a deficiency of a single complement regulatory protein emphasizes the largely non-redundant organization of complement control mechanisms. Once complement proteins are secreted by ECs or recruited from other cells to the surface of ECs, the classical complement effector system may be activated by several mechanisms. The binding of C1q to EC C1q receptors is a minor complement activation pathway, but the greater respective roles of C1q bound to EC surface C-Reactive Protein, pentraxin 3, phosphatidylserine or to antibodies on proteins such as class I HLA remain to be definitively elucidated. C5b-9 MAC is capable of injuring ECs directly by inducing membrane damage, whereas C3b injures ECs indirectly by attracting monocytes, enhancing their adherence to ECs and stimulating their diverse cytotoxic mechanisms.
