# Supplementary material for: Elevated complement mediator levels in endothelial-derived plasma exosomes implicate endothelial innate inflammation in diminished brain function of aging humans
Source: Sci Rep. 2021 Aug 10;11:16198. doi: 10.1038/s41598-021-91759-2 (PMC8355229; doi:10.1038/s41598-021-91759-2)
Supplement: Supplementary file 2 — Supplementary Information 2. [file 41598_2021_91759_MOESM2_ESM.pdf]

Title: Elevated complement mediator levels in endothelial-derived plasma exosomes implicate endothelial innate inflammation in diminished brain function of aging humans

Authors: Fanny M. Elahi[1*], Danielle Harvey[2], Marie Altendahl[1], Nivetha Brathaban[1], Nicole Fernandes[1], Kaitlin B. Casaletto[1], Adam M. Staffaroni[1], Pauline Maillard[3], Jason D. Hinman[4], Bruce L. Miller[1], Charles DeCarli[3], Joel H. Kramer[1], Edward J. Goetzl[5,6,7*]

[1]Memory and Aging Center, Department of Neurology, University of California, San Francisco. San Francisco, CA, USA

[2]Department of Public Health Sciences, University of California, Davis. Davis, CA, USA

[3]Department of Neurology and Center for Neuroscience, University of California, Davis. Davis, CA, USA

[4]Department of Neurology, University of California, Los Angeles. Los Angeles, CA, USA

[5]Department of Medicine, University of California, San Francisco. San Francisco, CA, USA

[6]Jewish Home of San Francisco. San Francisco, CA, USA

[7]Geriatric Research Center, 1719 Broderick St., San Francisco, CA, USA

Keywords: cerebral small vessel disease; exosomes; innate immunity; inflammation; complement factors; biomarkers; white matter; neurodegeneration.

[*]Corresponding Authors:
Fanny M. Elahi, MD PhD
675 Nelson Rising Lane, Suite 190, San Francisco, CA, 94158
Emails: fanny.elahi@ucsf.edu and elahilab15@gmail.com

Edward J. Goetzl, MD
Geriatric Research Center, 1719 Broderick St., San Francisco, CA 94115
E-mail: edward.goetzl@ucsf.edu

Supplemental Figure 2 | Nanoparticle Tracking Assay

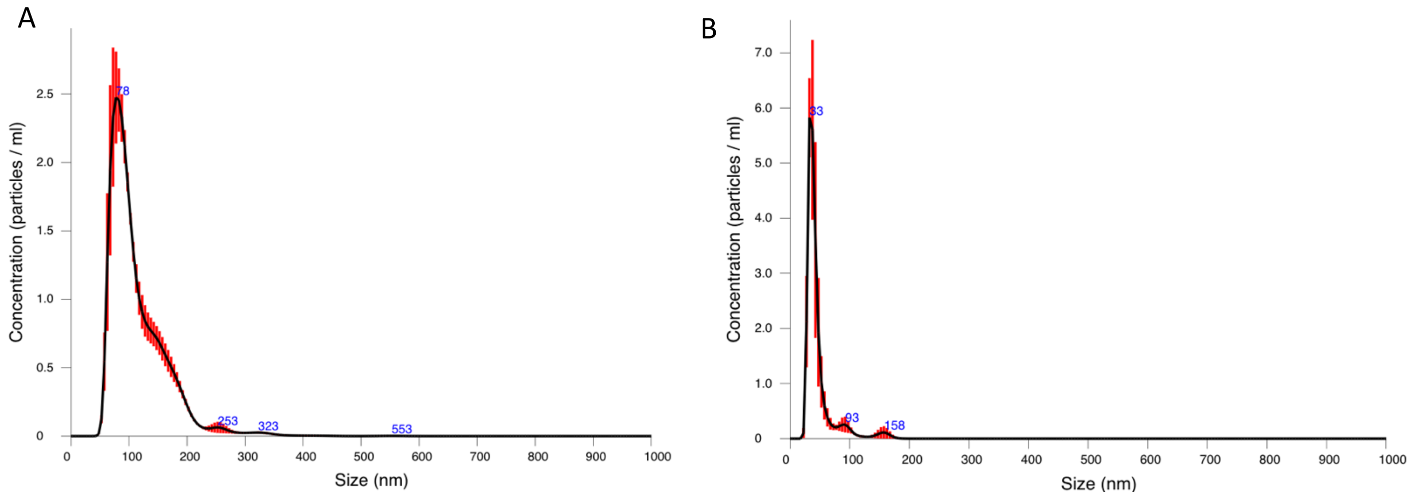

Results of NTA, without (A) and with (B) ExoGlow dye for typical EDE samples.
